# Successful removal of a thrombus in the setting of SVC syndrome using the INARI FlowTriever device

**DOI:** 10.1016/j.radcr.2021.12.032

**Published:** 2021-12-28

**Authors:** John M Sousou, Douglass M Sherard, Jamie R Edwards, Elsio Negron-Rubio

**Affiliations:** Department of Interventional Radiology, Ascension St. Vincent's Hospital, Jacksonville, FL, USA

**Keywords:** FlowTriever, ClotTriever, INARI, SVC syndrome, Pulmonary embolism, Thrombolytics, Mechanical thrombectomy

## Abstract

This case report describes a 56-year-old female who presented to the emergency department with diffuse facial and bilateral upper extremity swelling. The patient has a past medical history of Superior vena cava (SVC) syndrome secondary to a clot around her port-a-cath, adenocarcinoma of the lungs status post chemotherapy and radiation, hyperlipidemia, rheumatoid arthritis, diverticulitis status post colon resection, and hypothyroidism. Imaging confirmed the presence of a thrombus obstructing the SVC, likely due to her hypercoagulable state. This case report details the successful removal of a thrombus using the FlowTriever device by INARI in a patient with SVC syndrome. Although indicated for treatment of PE, FlowTriever has shown success in other conditions and nearly eliminates the risk of bleeding without the need for administering thrombolytics, as explained below in the setting of SVC syndrome.

## Introduction

Superior vena cava (SVC) syndrome is a result of obstruction of blood flow through the SVC, secondary to a thrombus, malignancy, or chest infection (ie, tuberculosis). In the United States alone, there are approximately 15,000 cases diagnosed each year [1]. The two predominating causes of SVC syndrome include malignant tumors (around 60% of cases) and thrombosis due to central lines, indwelling catheters, and pacemakers (30%-40% of cases) [2]. The cases due to thrombosis are rising due to the increasing usage of indwelling intravenous catheters and pacemakers, placing these patients in a hypercoagulable state. As a result of impaired drainage of blood from the head, neck, and upper extremities, patients experience symptoms of facial and neck swelling, upper extremity swelling, cough, shortness of breath, chest pain, and venous distension.

The use of thrombolytics has long been a novel and immediate therapy for patients with a deep vein thrombosis or PE. However, there is an increased chance of bleeding which may be a relative contraindication for usage in certain patients. The FlowTriever system by INARI is an FDA-approved device that was developed to nearly eliminate the risk of bleeding through the use of their own mechanical thromboembolectomy device without a need for administering thrombolytics [3]. The FlowTriever device allows for the removal of large volumes of blood clots while minimizing the amount of blood loss in the patient. FlowTriever is primarily used for the removal of a PE, however it may also be used for several other thrombus locations, such as in the setting of SVC syndrome as explained below.

## Case description

A 56-year-old female presented to the emergency department with diffuse facial and bilateral upper extremity swelling over the last two days. On exam, the patient denied any chest pain, shortness of breath, headache, dizziness, fever, coughing, nausea, and vomiting. The patient has a history of SVC syndrome secondary to a clot around her port-a-cath. She underwent angioplasty and thrombectomy of the distal right brachiocephalic vein/superior vena cava at the time with markedly improved anterograde flow to the right heart. She has a past medical history of adenocarcinoma of the lungs status post chemotherapy and radiation, hyperlipidemia, rheumatoid arthritis, diverticulitis status post colon resection, and hypothyroidism. Her initial vitals were stable at a blood pressure of 107/72 mm Hg, heart rate of 113 bpm, respiratory rate of 16 br/min, and an oxygen saturation of 97%. Initial lab results showed a high white blood cell count of 12.00 k/mcL, low hemoglobin of 10.9 g/dL, low hematocrit of 33.5%, platelet count of 301 k/mcL, Prothrombin time of 11.8 seconds, international normalized ratio of 1.0, and a high partial thromboplastin time of 38.1 seconds. Due to this patient's risk of hypercoagulability from her chemotherapy treatments and port-a-cath placement, the hematology/oncology department was contacted, who stated to admit the patient and have interventional radiology consulted for likely needing a thrombectomy.

Local anesthesia was administered, and an incision was made at the site of the indwelling port. The proximal catheter was cut and a stiff Glidewire was advanced to the superior vena cava. The catheter was then removed, and a 4 French MPA was advanced over the wire. The Glidewire and MPA were advanced into the right atrium, followed by removal of the Glidewire and advancement of an Amplatz wire into the inferior vena cava. A 6 French sheath was then placed over the wire, and contrast was injected which revealed high-grade stenosis of the superior vena cava with prominent venous collateral.

Right common femoral vein access was achieved through the use of ultrasound with a micropuncture set including a 21-gauge needle. The INARI FlowTriever was advanced over the wire to the superior vena cava where multiple aspirations of the thrombus were performed. These aspirations withdrew several fragments of white tissue, indicating a chronic thrombus. The INARI ClotTriever device was then used to mechanically retract the thrombus and improve antegrade flow. The wire and catheter were inserted into the right axillary vein. The ClotTriever device was advanced over the wire into the subclavian vein, and then retracted into the superior vena cava with successful removal of the thrombus. Post-thrombectomy venography revealed restoration of antegrade flow with a small degree of residual stenosis and decreased size of venous collaterals. A 6 cm x 10 mm Covera covered stent was deployed in the SVC across the stenosis. The stent was expanded with a 10 mm x 4 cm conquest balloon. Repeat venography performed through the sheath demonstrated a widely patent SVC. The guidewire and sheath were removed, and manual pressure was held until hemostasis was achieved. The patient tolerated the procedure well and there were no post-procedural complications.

## Discussion

**Fig. 1:**
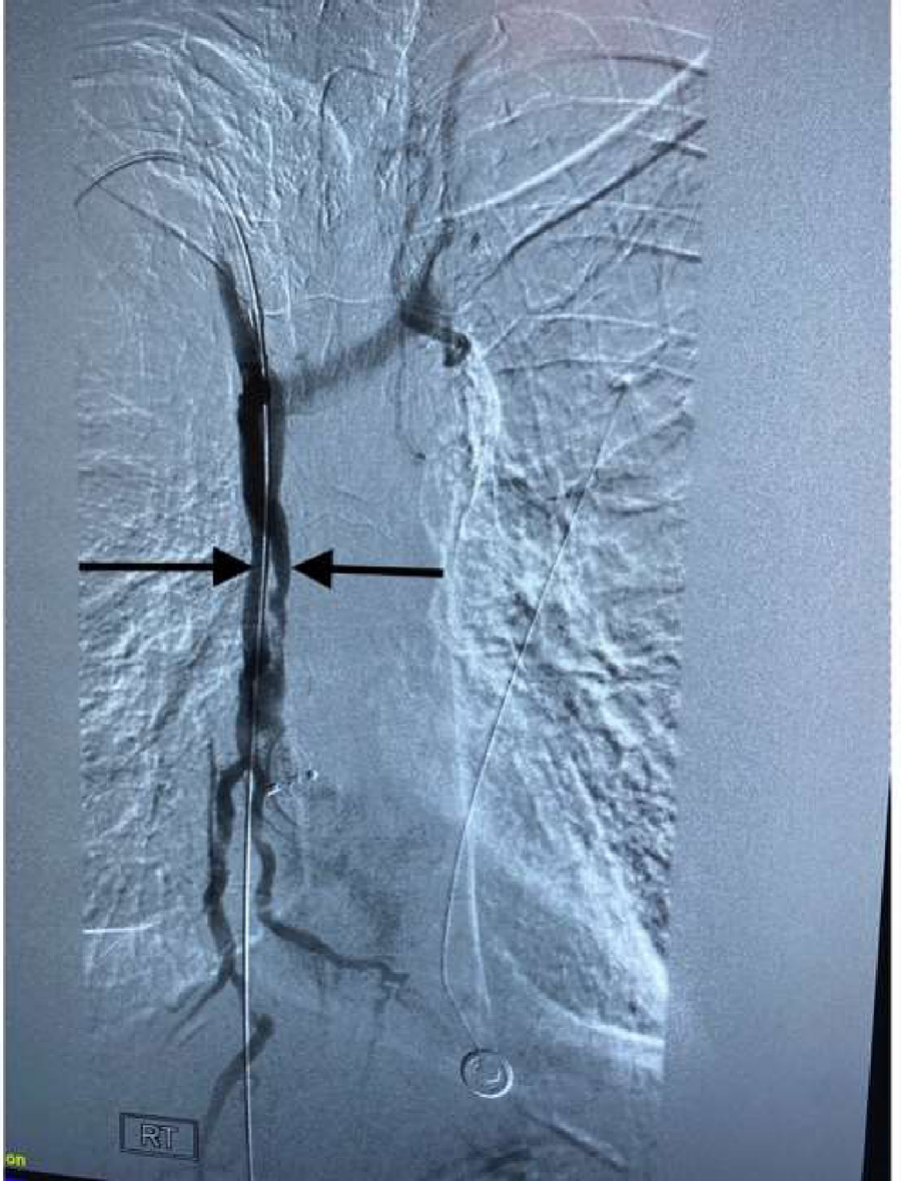
X-ray venography showing a thrombus obstructing the superior vena cava, as indicated by the yellow arrows.

**Fig. 2:**
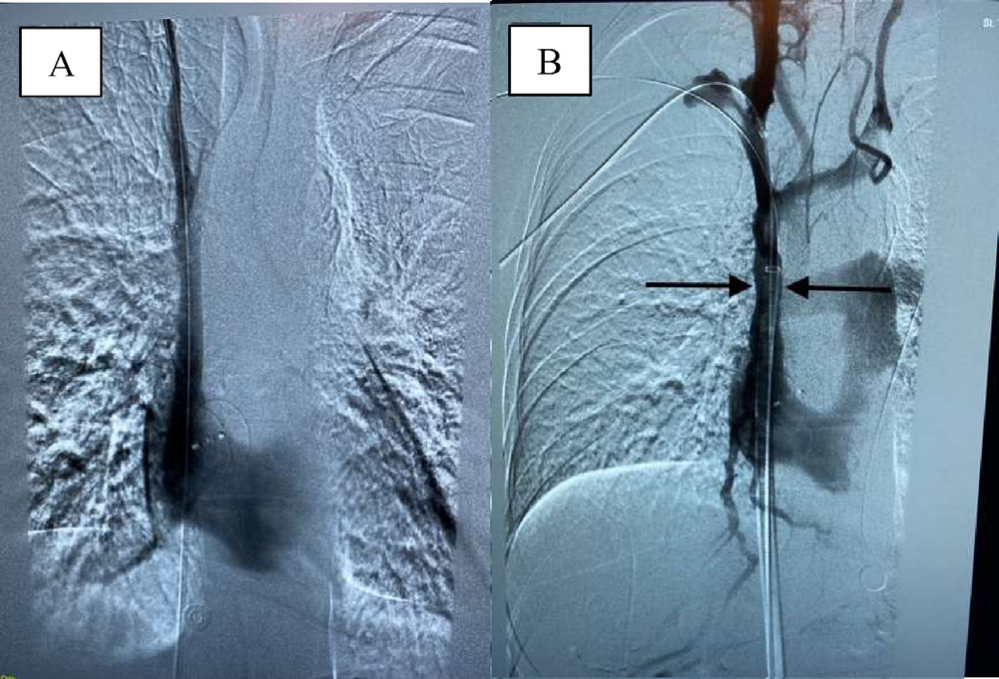
X-ray venography showing a non-obstructed SVC post-thrombectomy. A. Restoration of blood flow in the superior vena cava. B. Placement of a stent to prevent residual stenosis of the SVC.

**Fig. 3:**
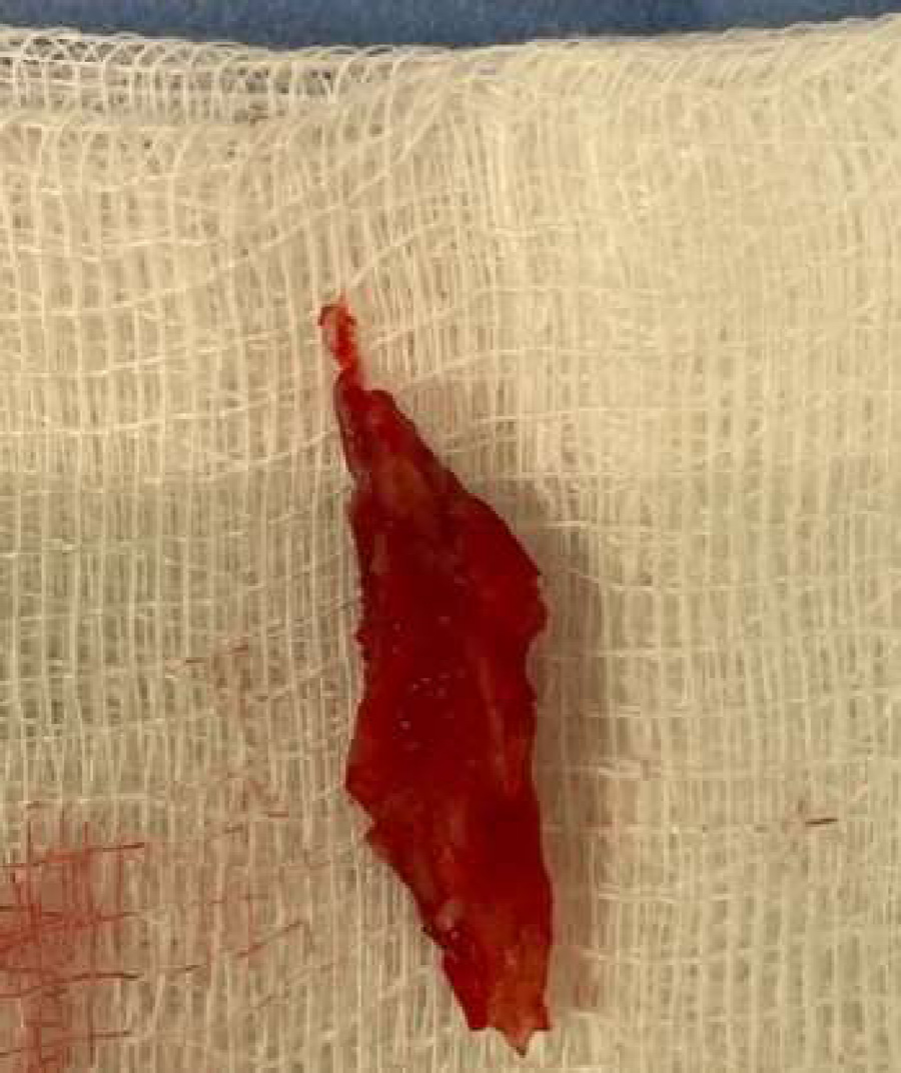
SVC thrombus extracted using the FlowTriever device by INARI.

**Fig. 4:**
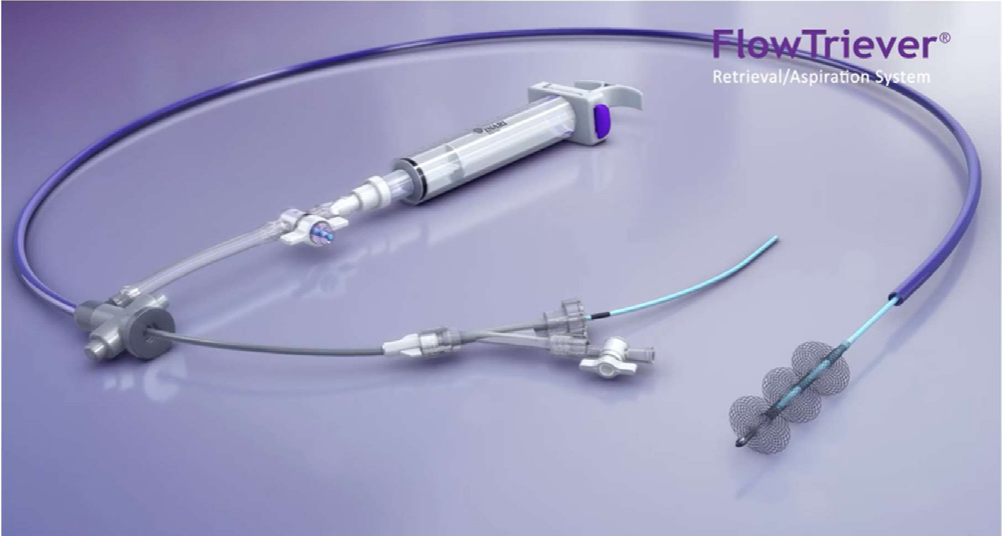
The INARI FlowTriever device [8].

This case details one of the first documented cases of the removal of a thrombus using the FlowTriever system by INARI in the setting of SVC syndrome. This unique case was successful as demonstrated by the rapid restoration of blood flow, elimination of symptoms, and no notable post-procedural outcomes. This patient also had no bleeding events following the procedure, as thrombolytic therapy was not needed, minimizing the risk of bleeding [4], [5], [6], [7], Figs. 1-4.

The INARI FlowTriever device is designed with a large lumen catheter, a large bore syringe, and three Nitinol mesh disks which are all used to retrieve the clot, while also minimizing the amount of blood lost during the procedure. After gaining venous access, the guidewire is inserted to the location of the thrombus and is then advanced past the clot. Once the clot is reached, the dilator is removed. The syringe then locks into place while the flow switch is turned off. The syringe is then pulled back and locked. Once the flow switch is turned on, this creates a vacuum-like suction which rapidly extracts the thrombus and delivers it into the syringe. The clot in the syringe is then pushed out onto a sterile gauze pad. If there is any clot remaining inside the vessel, the catheter is inserted through the clot and is then removed. Upon removal of the catheter, the Nitinol disks enlarge to the vessel walls and function to pull the rest of the clot out from the walls of the vessels and into the FlowTriever device, which is then removed from the patient [8].

The FlowTriever system has been proven to be an efficient modality for mechanical thrombectomies in the setting of PE, however success has also been shown by both the ClotTriever and FlowTriever systems in conditions other than PE. For example, a study by Murali et al demonstrated the success of the FlowTriever system in patients with IVC thrombosis, showing no postoperative complications of re-thrombosis, renal damage, myocardial infarction, intensive care unit stay, or death [9]. Additionally, these patients did not require the use of thrombolytics, minimizing their risk of bleeding events. Another study detailed the successful use of FlowTriever for the removal of a thrombus from the right atrium of a patient with transthoracic echocardiogram guidance [10].

The FLARE study in 2019 displayed the safety and efficacy of the FlowTriever system in patients with acute intermediate-risk PE [11]. They found significant improvement in ventricular function, minimal bleeding complications due to absence of thrombolytics, and reduced need for post-procedural critical care. Certain studies have demonstrated the use of FlowTriever outside the scope of PE, however more studies like these are needed to further investigate the efficacy of the system as a potential alternative to thrombolytics and surgical intervention.

## Conclusion

The FDA-approved FlowTriever system has proven to be a successful alternative treatment for patients with PE. Other studies have also shown success of the FlowTriever system outside the scope of PE, such as this case in the setting of SVC syndrome. More studies however are needed to further determine the safety and efficacy of FlowTriever in conditions outside of PE, which could potentially offer additional treatment options without the risks associated with thrombolytic therapy and surgical intervention.

## Patient consent

Consent from the patient was not attained for the publication of this case report since there are no patient identifying characteristics included in the report.
